# Hybrid Deep Learning Framework for Continuous User Authentication Based on Smartphone Sensors

**DOI:** 10.3390/s25092817

**Published:** 2025-04-30

**Authors:** Bandar Alotaibi, Munif Alotaibi

**Affiliations:** 1 Department of Information Technology, University of Tabuk, Tabuk 47731, Saudi Arabia; 2 Dahaa Research Group, Department of Computer Science, Shaqra University, Shaqra 11961, Saudi Arabia

**Keywords:** cybersecurity, wearable sensors, continuous user authentication, deep learning, behavioral patterns

## Abstract

Continuous user authentication is critical to mobile device security, addressing vulnerabilities associated with traditional one-time authentication methods. This research proposes a hybrid deep learning framework that combines techniques from computer vision and sequence modeling, namely, ViT-inspired patch extraction, multi-head attention, and BiLSTM networks, to authenticate users continuously from smartphone sensor data. Unlike many existing approaches that directly apply these techniques for specific recognition tasks, our method reshapes raw motion signals into ViT-like patches to capture short-range patterns, employs multi-head attention to emphasize the most discriminative temporal segments, and then processes these enhanced embeddings through a bidirectional LSTM to integrate broader contextual information. This integrated pipeline effectively extracts local and global motion features specific to each user’s unique behavior, improving accuracy over conventional Transformer, Informer, CNN, and LSTM baselines. Experiments on the MotionSense and UCI HAR datasets show accuracies of 97.51% and 89.37%, respectively, indicating strong user-identification performance.

## 1. Introduction

Mobile devices are essential for communication and access to sensitive information [1]; however, this reliance raises security concerns about user identification and session continuity [2]. Traditional authentication methods, such as PINs, passwords, and biometrics, provide an initial layer of security but fail to offer continuous protection throughout a session [3]. This limitation creates an opportunity for unauthorized access, for example, if a device is left unattended or compromised after the initial authentication. Static methods usually do not adapt efficiently to changing threats, leaving devices exposed if the attacker gains access to them after the initial login. This issue has created a need for more adaptive methods of verifying user identity.

Continuous authentication, which involves verifying the user’s identity in real time while the device is in use, has emerged as a promising solution to these concerns [4]. Unlike conventional methods, continuous authentication leverages behavioral and physiological features, offering an unobtrusive and dynamic security mechanism [5]. Among the various behavioral metrics available, power consumption patterns [6], touch gestures [7], and physical movements [8] have shown great potential as unique identifiers for distinguishing legitimate users from intruders, enabling the detection of unauthorized device usage with high accuracy in a short amount of time. In particular, smartphone sensors (e.g., accelerometers, gyroscopes, and magnetometers) enable the real-time measurement of user motion and interaction patterns, yielding highly individualized profiles that can serve as continuous authentication signatures. Smartphone sensor readings can also be viewed as behavioral biometrics because they capture an individual’s distinctive motion patterns [9]. Multiple studies have shown that users demonstrate unique gait signatures, micro-movements, or interaction styles that remain relatively stable within individuals while varying significantly among them [10]. For instance, differences in stride length, device grip force, and hand tremors collectively serve as a personal motion fingerprint. These fine-grained distinctions are not purely random noise; they arise from each user’s neuromuscular coordination, posture, and habitual interaction style. As a result, motion-based signals have been recognized as sufficiently consistent and discriminative to meet the criteria for biometric traits, namely, universality, uniqueness, permanence, and collectability, making them viable for continuous authentication [11]. There are various challenges associated with developing a reliable continuous authentication system, such as extracting local and global motion signatures. Several approaches concentrate on short-term features, such as high-level statistical patterns and sliding windows, which can neglect important temporal dependencies across user actions. Second, model architectures that rely on simpler pipelines can underfit the complexity of the data or are not flexible enough to integrate various sensor modalities. Third, many approaches struggle to adapt seamlessly across diverse user contexts (e.g., standing, walking, changing, and device orientation), impacting real-time performance.

Modern authentication paradigms emphasize one-time credential checks and continuous verification in line with evolving zero-trust principles and passwordless approaches [12,13]. Moreover, recent studies have leveraged advanced sensor analysis and deep learning models to deliver real-time authentication that adapts to diverse usage conditions [14,15]. Despite progress in biometrics and behavior-based verification, many solutions still face challenges in robustly capturing dynamic user activity across multiple sensors and contexts. The present work proposes a hybrid deep learning framework combining local-patch extraction, multi-head attention, and bidirectional sequence modeling to address these gaps. By extending current deep-learning-based methods, we demonstrate that carefully integrated architectures can better adapt to changing motion signatures, ultimately improving authentication reliability.

Recent advances in deep learning provide powerful techniques for processing complex data, including sensor signals. In particular, CNNs effectively learn local patterns, while transformers utilize a multi-head attention mechanism to capture long-range dependencies [16]. LSTM and BiLSTM architectures also improve sequence modeling by retaining important temporal context through gating mechanisms [17]. ViTs adapt the transformer concept to image patches [18] and can similarly be applied to sensor patches [19]. These components act as flexible building blocks in a hybrid pipeline that integrates localized feature extraction (using CNN-like or ViT-like modules), global attention (through multi-head attention), and temporal sequence learning (via BiLSTM). By combining these established techniques, our approach takes advantage of their strengths in local and global representation learning and forward and backward context without extensive manual feature design.

This study contributes to the field of mobile security by showing how motion sensor data and user interactions can be combined into a practical continuous authentication system. We show that a hybrid deep learning approach, using vision transformer (ViT)-inspired patch extraction, multi-head attention, and bidirectional LSTM modeling, can effectively capture user-specific motion signatures while overcoming the limitations of single-sensor and conventional machine learning methods. Single-sensor approaches often yield a narrow perspective on user behaviors by focusing on only one type of signal (e.g., from an accelerometer), thus missing complementary information from other data sources, such as gyroscopes or magnetometers.This limited coverage can lead to lower accuracy and poorer robustness when user behavior varies, such as walking versus jogging. Similarly, conventional machine learning methods typically rely on hand-crafted features or simpler models that do not fully capture the temporal complexity and interdependencies of sensor streams. Our proposed hybrid framework addresses these gaps by combining multiple sensors (accelerometers, gyroscopes, and possibly others) to provide a richer representation of user motion. In addition, the framework automatically extracts features through a patch-based approach inspired by vision transformers, reducing the need for manually engineered features. Lastly, the architecture leverages multi-head attention and BiLSTM layers to capture global (long-range) and local (short-term) dependencies in time-series data, capabilities that simpler models often lack. When combined, these elements enable local pattern extraction, global relational modeling, and sequential context in a unified framework, offering advantages over simpler architectures. This design boosts accuracy and enhances resilience to different user activities and device orientations, addressing the limitations of single-sensor and traditional machine learning solutions. The proposed approach can be expanded to include other wearable technologies like electrocardiograms (ECGs) and photoplethysmographs (PPGs), enhancing motion-based behavioral profiles with physiological data. By creating a modular and adaptable system, we anticipate a future of continuous, convenient smartphone authentication that monitors user behavior in real time.

This paper is structured as follows: In Section 1, the Introduction outlines the motivation, challenges, and scope of continuous user authentication using smartphone sensor data. This is followed by a review of the related literature in Section 2, highlighting existing methodologies and their limitations. The research methodology is presented in Section 3, which explains the design and implementation of the hybrid deep learning model, including data collection and preprocessing (Section 3.1) and the mathematical framework (Section 4). Next, the evaluation and validation in Section 5 provide metrics, experimental protocols, and performance analysis, supported by the results in Section 5.3, such as validation and loss curves and confusion matrices. Finally, conclusions are provided in Section 6, which summarizes the key findings, their implications, and future research directions, ensuring a comprehensive and structured narrative.

## 2. Related Work

Sagbas and Balli [14] presented a method for enhancing smartphone security by continuously verifying the user’s identity via behavioral biometrics. The authors analyzed users’ typing behaviors on soft keyboards and used data from motion sensors to develop a continuous authentication system. The study involved 59 participants, from whom 125 unique features were extracted, encompassing accelerometer and gyroscope readings as well as touchscreen interactions. A correlation-based feature selection (CFS) method was employed to identify the most suitable features. Subsequently, the data were classified using random forest (RF), k-nearest neighbors (kNN), and logistic regression (LR) models. The proposed system achieved a classification accuracy of up to 93%, with user identification processing times as low as 0.03 milliseconds. Although 93% accuracy is moderate, it remains below the performance thresholds often targeted by more recent deep learning architectures for continuous authentication, which can reach more than 93% under comparable conditions. Moreover, the reliance on classical machine learning models may limit the method’s ability to capture the more subtle temporal and contextual relationships present in real-world situations.

Alzahrani et al. [15] introduced a novel approach to continuous and passive mobile user authentication, leveraging smartphone sensors and deep learning. The proposed system combines a convolutional neural network (CNN) and a bidirectional long short-term memory (Bi-LSTM) network to authenticate users based on their physical activities. Their experiments showed significant improvements over traditional machine learning, highlighting the promise of integrated deep-learning architectures for continuous authentication. However, their approach mainly uses raw sensor data without specialized segmentation strategies or advanced attention mechanisms. The CNN component extracts basic local features, and although BiLSTM can handle a forward-and-backward temporal context, it does not incorporate any mechanism such as multi-head attention to learn which parts of the sequence are most discriminative for user identification, patch-based feature embedding, or explicit modeling of global interdependencies across sensor windows. This can become limiting when user activities vary significantly (e.g., transitioning between walking, running, and sitting) as the model may underutilize relevant sensor segments that appear intermittently or at irregular intervals.

Centeno et al. [20] proposed a continuous authentication system using a Siamese convolutional neural network to analyze motion patterns. The system used a public human activity dataset and achieved a verification accuracy of up to 97.8%. The method is noted for its robustness to sampling frequency and sequence length variations. Their approach measures the similarity of feature embeddings between pairs of motion sequences, effectively deciding whether two samples originate from the same user. However, the pairwise comparison nature of Siamese networks may not scale efficiently to more significant multi-class user identification scenarios in which the system must discriminate among many subjects rather than merely verify whether two samples come from the same individual.

Abuhamad et al. [21] introduced a deep-learning-based continuous authentication system called AUToSen that utilizes behavioral data from smartphone sensors. It identifies users implicitly by capturing and analyzing patterns in accelerometer, gyroscope, and magnetometer data. The system achieved an F1-score of 98% with low rejection rates. However, AUToSen primarily relies on a a binary classification approach. For each user, the system learns to distinguish between a legitimate user and others rather than simultaneously identifying which of many possible users produced the motion data. This setup can be less demanding than a true multi-class classification scenario in which every sample must be matched to its correct user ID among a larger set of subjects.

Mekruksavanich and Jitpattanakul [8] introduced a framework called DeepAuthen, which uses deep learning classifiers. This model aims to identify smartphone users based on patterns of physical activity captured using accelerometer, gyroscope, and magnetometer sensors, highlighting the effectiveness of integrating motion sensors in continuous authentication systems. However, while DeepAuthen integrates multiple sensor modalities, its binary design does not address scenarios in which a system must identify which user among several is holding the device, as is often required in larger-scale multi-user deployments.

Cao et al. [22] presented a hybrid deep learning model that combines CNN and LSTM networks for user authentication based on gait. The model processes gait data collected from smartphone sensors to recognize user identity, achieving improved performance metrics compared to traditional methods. However, their work focuses on gait signals alone, which may not fully capture diverse user behaviors arising from other activities such as jogging, climbing stairs, or interacting with the device in varied postures.

Centeno et al. [23] explored the application of deep learning autoencoders for continuous user authentication on smartphones. They focused on identifying behavioral patterns to enhance security, achieving high classification accuracy while minimizing computational overhead. However, their approach treats the authentication problem as an anomaly detection task (i.e., a legitimate vs. fraudulent user), which can be less comprehensive than an actual multi-class setting in which multiple users must be distinguished.

Wang et al. [24] presented a continuous authentication framework combining multimodal biometric data, such as touch behavior and sensor data. They employed a CNN–LSTM–Attention algorithm to analyze user behaviors and classify activity patterns. The approach is memory-efficient and delivers better performance compared to traditional classification models. However, they focus on combining touch and motion signals (e.g., touchscreen interactions and basic motion data) and do not fully explore finer segmentation approaches for time-series data. Additionally, the attention mechanism described is generally applied to the output of the LSTM without employing a patch-based strategy or multi-head attention that could capture more detailed local and global dependencies across diverse sensor inputs.

Li et al. [25] introduced SNNAuth, a continuous authentication framework that leverages smartphone sensor data and spiking neural networks (SNNs). The system extracts behavioral patterns from accelerometer and gyroscope data, offering a highly discriminative and energy-efficient solution for continuous authentication. However, the framework is designed primarily for binary classification, distinguishing a single legitimate user from potential impostors rather than identifying which user among many is holding the device. While this simplified setup can still yield high accuracy with low power consumption, it does not address the more complex scenario in which the system must simultaneously discriminate among multiple enrolled subjects.

Shen et al. [26] introduced MMAuth, which combines a CNN and LSTM to perform continuous authentication by analyzing multimodal data from smartphone sensors. In this model, the CNN captures spatial patterns, while the LSTM processes temporal dependencies, enabling accurate user authentication based on activity and behavioral data. Although the authors integrated a CNN and LSTM for feature extraction, the final classification step employs a deep-learning-based one-class classifier. This setup effectively verifies one legitimate user against impostor behavior rather than distinguishing among multiple enrolled subjects. As a result, the framework is considered a one-class classifier, focusing on detecting whether incoming signals match the owner’s profile. In contrast, our proposed hybrid approach handles multi-class classification in which each subject represents a separate class. This multi-subject setting is more complex because the system must identify exactly which user out of many produced the sensor stream rather than simply deciding whether a sample comes from a legitimate user or an impostor.

Yantao et al. [27] proposed CAGANet, a CNN-based continuous authentication system enhanced with a conditional Wasserstein generative adversarial network (CWGAN). The CWGAN is used for data augmentation, thus improving the model’s robustness, and handling variability in user behavior. After extracting features through a CNN, they trained four one-class classifiers (i.e., one-class-SVM, LOF, isolation forest (IF), and elliptic envelope (EE)) during the enrollment phase. This one-class approach indicates that each user is modeled separately, verifying whether the incoming sensor data match or deviate from their learned motion profile, thus distinguishing legitimate users from impostors. While this approach can yield high accuracy and robustness (due in part to the CWGAN-augmented data), it does not tackle a multi-class identification scenario in which the system must determine which user, among many, is holding the device.

Muaaz et al. [28] introduced a novel IntelliAuth framework designed to enhance smartphone security through continuous user authentication. The authors propose leveraging the behavioral characteristics and context of users—specifically, their physical activities and surrounding environment—as biometric indicators for authentication. The IntelliAuth framework utilizes data from embedded smartphone sensors, including accelerometer, gyroscope, and magnetometer data, to recognize six daily activities: walking, sitting, standing, running, and walking up and down stairs. The system also considers five common smartphone positions on the user’s body during these activities. The framework identifies unique patterns corresponding to each user by analyzing sensor data. The study employed a dataset for the recognition of physical activity, from which features were extracted in both the time and frequency domains. These features were then classified into the six defined activities that different users perform. The proposed framework also provides a multi-class smart user authentication platform, offering varying levels of access to a wide range of smartphone users. Through a series of experiments, the effectiveness of the IntelliAuth framework in accurately recognizing user activities and distinguishing between users based on their behavioral patterns was demonstrated. This approach offers a promising solution for continuous and passive authentication, enhancing the security of mobile devices without compromising user convenience. While IntelliAuth’s use of activity context and variable device positioning is beneficial for capturing diverse real-world behaviors, the framework relies on more conventional feature extraction approaches rather than an end-to-end deep learning pipeline. Furthermore, the authors only compare well-known classifiers to evaluate their extracted features instead of exploring more advanced deep architectures. Consequently, IntelliAuth typically employs classical measures (e.g., statistical or frequency-based features) and established classification algorithms to distinguish among users.

Sánchez et al. [29] presented AuthCODE, an architecture for continuous authentication across multiple devices that leverages machine and deep learning. It focuses on preserving privacy while analyzing user behavior in smart offices. AuthCODE integrates behavioral data from various devices, achieving improved authentication accuracy. However, their approach focuses more on multi-device integration and privacy-preserving analytics than advanced time-series feature extraction from smartphone sensors alone.

Cariello et al. [30] propose a framework that detects change-of-possession events on a smartphone (e.g., another user grabbing the device) and then triggers re-authentication. Unlike standard continuous authentication, which periodically checks user identity at fixed intervals, this approach monitors accelerometer and gyroscope data to identify precisely when the legitimate user no longer has possession of the device. This can potentially reduce both false re-authentication prompts and the time window in which an impostor can misuse an unlocked phone. However, their method focuses on discrete transition events, which may not effectively capture long-duration or subtle motion patterns. While it can identify short changes in possession, additional modeling may be needed for more continuous or diverse user behaviors.

Lee and Lee [31] proposed an implicit authentication system that leverages behavioral characteristics captured with smartphone sensors for continuous user identity verification. The system employs machine learning techniques to analyze data from accelerometers, gyroscopes, and other sensors and achieved an authentication accuracy of 98.1%. The study demonstrated the feasibility of continuous authentication methods that operate seamlessly in the background, enhancing security without compromising user convenience. However, it relies on a smaller, private dataset with fewer participants and focuses on simpler machine learning pipelines. Our proposed framework draws on larger open datasets and integrates advanced deep learning mechanisms. This shift toward more complex yet robust architectures is better suited for modeling user motion signatures at scale, capturing local and global sensor data dependencies. Direct performance comparisons are not straightforward as their methodology and dataset size differ significantly from our hybrid approach and used datasets. A comparison of our method and the related work is shown in Table 1.

## 3. Dataset Information

This research focuses on designing and evaluating a hybrid deep learning model for time-series classification, aiming to differentiate which user produced a given sensor stream in the context of continuous authentication. Specifically, we combine elements from ViT, multi-head attention mechanisms, and BiLSTM networks. Our experiments focus on identifying 23 subjects using the MotionSense dataset and 9 subjects using the UCI HAR dataset. Below, we describe the key components of our methodology—namely, data collection and preprocessing, followed by a new section about formulating the model—and highlight how these processes interconnect in our overall experimental design.

### 3.1. Data Collection and Preprocessing

In this study, we used two different datasets, each representing a variety of human motion patterns that can be utilized for subject identification for continuous authentication. The essential attributes of these datasets—MotionSense [32] and UCI HAR [33]—are summarized in Table 2. Detailed descriptions and the specific preprocessing steps for each dataset are presented in the following subsections.

#### 3.1.1. MotionSense Dataset

MotionSense was originally assembled to classify human activities; in this study, it was re-purposed for subject identification in a continuous authentication framework. The data were captured using an iPhone 6S placed in the participant’s front pocket using the SensingKit framework [32]. A total of 24 participants (14 males and 10 females) performed six activities (walking down stairs, walking up stairs, walking, jogging, sitting, and standing) in 15 trials under uniform conditions. Rather than classifying which activity each participant performed, we focus on who performed it, re-framing the dataset to study motion-based user signatures.

MotionSense encompasses three core sensor modalities:

- Accelerometer: captures acceleration (including gravity);
- Attitude: measures orientation angles (pitch, roll, and yaw);
- Gyroscope: records angular velocity.

All 12 features (m=12) were sampled at 50 Hz. As activity recognition is incidental to our goal, these signals are primarily used to identify distinct individuals. Detailed documentation and code for these data can be accessed from [32]. As noted in Table 2, one participant (ID 7) lacked sufficient samples, leaving 23 subjects with adequate data.

Preprocessing began by confirming the integrity of the data and removing trials that were corrupt or incomplete. We then segmented the motion data into short windows (e.g., $\left(\mathrm{batch},\;n_{s}\times n_{l},\;n_{f}\right)$), normalizing each feature for consistent ranges. Finally, each window was assigned a subject ID rather than an activity label, considering the subject-identification objective. Through re-interpreting the MotionSense dataset in this manner, we reveal how user-specific motion signatures persist across various everyday tasks and conditions.

#### 3.1.2. Human Activity Recognition (HAR) Using Smartphones (UCI)

We also employed the UCI HAR dataset [33], which was originally intended for activity recognition but is equally suitable for investigating subject identification. It includes 30 volunteers (ages 19–48), each performing six daily activities (standing, sitting, lying down, walking, walking down stairs, and walking up stairs). An Android smartphone’s accelerometer and gyroscope were used to capture the data at 50 Hz. To reduce noise, a median filter and a third-order low-pass Butterworth filter (cutoff at 20 Hz) were applied during data acquisition, further separating body and gravity signals. Windows with length of 2.56 s (with 50% overlap) were used, guaranteeing at least one stride cycle for typical walking. The dataset additionally provides 561 engineered features, as summarized in Table 2.

To adapt the dataset for user identification, we relabeled each segment with the subject ID. However, as some subjects had very few samples, we retained nine individuals who produced sufficient data for robust training and evaluation. The public 70–30 training–testing split was refined to ensure that we could measure the model’s ability to generalize to unseen subjects. As with MotionSense, we standardized or normalized each feature (pitch, roll, yaw, etc.) for consistent scaling, optionally merging all channels into $\left(\mathrm{batch},\;n_{s}\times n_{l},\;n_{f}\right)$.

Changing the focus of the UCI HAR dataset from activity classification to subject identification provides a new context for testing continuous authentication. The diversity of the dataset, which includes various stationary postures, walking, and climbing stairs, illustrates the significant differences in motion data among participants. This emphasizes the importance of modeling attributes specific to each user. Ultimately, strong performance in both datasets indicates the broad utility of our vision transformer-like approach, multi-head attention, and BiLSTM structure for continuous user authentication.

#### 3.1.3. Overall Preprocessing Workflow for Both Datasets

MotionSense and UCI HAR were processed using the same settings, despite their differences in sensor specifications and initial purpose. We first segmented the time series into fixed-length windows, normalized features, and labeled each window by subject ID. The re-shaped samples were then fed to our deep learning pipeline, where vision transformer-like patches, multi-head attention, and a BiLSTM collectively learned user-specific features. Table 2 consolidates the main dataset characteristics, reflecting how each dataset contributed unique motion patterns for the subject identification experiments. The key hyperparameters and regularization methods used in training for both datasets are as follows. Each input sample was normalized via a standard scaler and then reshaped into a three-dimensional tensor suitable for the hybrid model. We initialized our model parameters using the default Keras initialization schemes for the Conv1D, Dense, and LSTM layers. The Adam optimizer was employed at a learning rate of 0.001 to balance rapid convergence with stable updates, and we trained for 282 epochs (for the MotionSense dataset) and 50 epochs (for the UCI HAR dataset). During training, early stopping (patience = 20) was applied to halt training when the validation loss stopped improving, preventing overfitting and reducing unnecessary computation. Model checkpoints further ensured that only the best-performing weights were retained. We incorporated dropout layers in the ViT and BiLSTM components. We set a recurrent dropout in the BiLSTM to mitigate overfitting and encourage generalization. Together, these choices reflect a balance of computational feasibility, model complexity, and robustness to overfitting for the continuous authentication task.

## 4. Proposed Method

This section details the theoretical backbone of our hybrid deep learning framework for continuous authentication. The model combines a ViT module, multi-head attention block, and BiLSTM layer. The ultimate goal was to identify which subject produced a given time-series segment by learning distinctive motion signatures.

### 4.1. Notation

Let *N* be the number of time-series windows (samples) in the dataset. Define $T=n_{s}\times n_{l}$ as the total number of time points in each window, where $n_{s}$ is the number of sub-sequences (or chunks), and $n_{l}$ is the length of each chunk. Let $F=n_{f}$ denote the number of features (e.g., different sensor axes) per time point. Hence, each sample can be expressed as a matrix $X_{i}\in R^{T\times F}$, where *i* indexes the sample from 1 to *N*. We assume $\lambda_{i}\in \left\{0,1,\ldots ,C-1\right\}$ is the integer label for the *i*th sample, with *C* being the total number of classes (i.e., the number of subjects to be identified). We denote the entire set of trainable parameters in the model by Θ, including convolution filters in the ViT, attention weights in the multi-head attention block, and recurrent weights in the LSTM layers. Once the model outputs a probability vector $p_{i}$ for sample *i*, the predicted class label is ${\hat{\lambda }}_{i}=\arg\max\left(p_{i}\right)$. Throughout this work, ℓ(·) denotes the chosen loss function. In our implementation, we employ sparse categorical cross-entropy, which is particularly suitable for multi-class problems with integer labels.

### 4.2. Problem Formulation

We aimed to learn a mapping function $f:R^{T\times F}\to \left\{0,1,\ldots ,C-1\right\}$ that accurately identified the correct subject from each time-series segment. Formally, our objective was to minimize

(1) $$\min_{\Theta }\;\;\ell \left(\Theta \right),\;\mathrm{where}\;\ell \left(\Theta \right)=-\frac{1}{N}\sum_{i=1}^{N}\log\left(p_{i,\lambda_{i}}\right),$$

and $p_{i,\lambda_{i}}$ is the predicted probability (the $\lambda_{i}$th component of $p_{i}$) corresponding to the true subject label $\lambda_{i}$. We trained the model via mini-batch gradient-based optimization, adjusting the parameters Θ to reduce the discrepancy between predictions and ground-truth labels.

### 4.3. Vision-Transformer-like Feature Extraction

To adapt the vision transformer concept to time-series data, we first split or patched the temporal sequence using a 1D convolutional operator. Specifically, we convolved each $X_{i}$ with filters $W_{c}$, added a bias $b_{c}$, and applied a nonlinear ReLU activation σ(·). Mathematically, this step can be summarized by

(2) $$Z_{\rho }=\sigma \left(X_{i}*W_{c}+b_{c}\right),$$

where ρ denotes the local patch concept, and ∗ is the convolution operation. The resulting local segments $Z_{\rho }$ are then passed through a flatten operator F(·) and a dense layer with weights $W_{d}$ plus bias $b_{d}$, followed by a dropout D(·) and another ReLU activation:

(3) $$z_{v}=D\left(\sigma \left(W_{d}\;F\left(Z_{\rho }\right)+b_{d}\right)\right),$$

yielding a feature vector $z_{v}\in R^{D}$, where *D* is the desired embedding dimension (e.g., 256). Via analogy to the vision transformer, these ρ-based embeddings capture local structure in the time series.

### 4.4. Multi-Head Attention for Temporal Modeling

After obtaining ViT-like embeddings, we introduced multi-head attention to learn temporal dependencies across the sequence. Each time-series sample $X_{i}$ was transformed into three sets of vectors: queries *G*, keys *J*, and values *E*. Each set was computed by multiplying $X_{i}$ by the corresponding parameter matrices $W_{G}$, $W_{J}$, and $W_{V}$, respectively. Symbolically,

(4) $$G=X_{i}\;W_{G},\;J=X_{i}\;W_{J},\;E=X_{i}\;W_{E},$$

where these weight matrices are learnable parameters specific to each head. The attention mechanism then computes a weighted combination of *E*, with weights derived via the scaled dot product of *G* and *J*

(5) $$A\left(G,J,E\right)=S\left(\frac{G\;J^{T}}{\sqrt{d_{j}}}\right)\;E,$$

where S(·) is the softmax, and $d_{j}$ is the dimension of keys and queries. In a multi-head setting, multiple such attentions $h_{1},h_{2},\cdots ,h_{H}$ are run in parallel (*H* heads). Their outputs are concatenated and projected by $W_{O}$, yielding

(6) $$M=\left[h_{1};\cdots ;h_{H}\right]\;W_{O}.$$

A residual connection often bypassed this projection, and we subsequently applied a layer normalization L(·) plus a feed-forward block, producing the final MHA output:

(7) $$M_{fin}=L\left(D\left(\sigma (M_{n}\;W_{1}+b_{1})\right)\right),$$

where $M_{n}=L\left(M\right)$ denotes the normalized *M*. The function σ(·) again denotes a ReLU or similar nonlinearity, while D(·) denotes dropout.

### 4.5. Bidirectional LSTM and Final Classification

To combine the complementary strengths of ViT embeddings and multi-head attention outputs, we first replicated $z_{e}$ across *T* time steps using a repeat operator R(·). This ensured that its dimensionality matched $M_{fin}$. We then concatenated these representations into a unified sequence:

(8) $$Z_{c}=\left[\;M_{fin};R\left(z_{e}\right)\;\right].$$

Subsequently, we fed $Z_{c}$ into a bidirectional LSTM layer, denoted as B(·), which processed the sequence forwards and backwards in time:

(9) $$\vec{h_{t}},\;\overset{\leftarrow }{h_{t}}=B\left(Z_{c}\right),$$

The resulting hidden states were concatenated to form a final context vector $h_{\mathrm{bi}}$. This vector captured temporal information from both directions, making it especially powerful for identifying subtle user-specific patterns. A dense layer with weights $W_{2}$ and biases $b_{2}$ then output class probabilities:

(10) $$p_{i}=S\left(W_{2}\;h_{bi}+b_{2}\right),$$

where S(·) again denotes the softmax function, and ${\hat{\lambda }}_{i}=\arg\max\left(p_{i}\right)$ denotes the predicted subject.

### 4.6. Loss Function and Optimization

Our model was trained using the sparse categorical cross-entropy loss:

(11) $$\ell \left(\Theta \right)=-\frac{1}{N}\sum_{i=1}^{N}\ln\left(p_{i,\lambda_{i}}\right),$$

where $p_{i,\lambda_{i}}$ is the probability assigned to the true subject $\lambda_{i}$.

We used the Adam optimizer with a learning rate of 0.001, which adaptively adjusted each parameter’s learning rate in Θ. Training proceeded via mini-batch gradient-based updates until the validation performance converged or an early stopping criterion was met. Through this process, the hybrid framework—consisting of ρ-based ViT embeddings, multi-head attention, and bidirectional recurrent modeling—learned distinctive user signatures for continuous authentication.

### 4.7. Key Implementation Details

**Figure 1:**
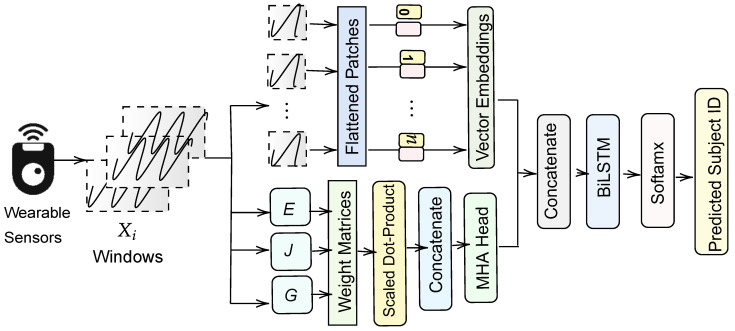
Our proposed hybrid deep learning model.

Each input sample was re-shaped to (batch,T,F) such that the vision transformer module and multi-head attention could process time-series data in a three-dimensional format. Standard callbacks such as early stopping and model checkpoints managed overfitting and selected optimal weights automatically. Early stopping halted training if the validation loss failed to improve for a specified number of epochs, while a model checkpoint preserved the best model whenever a new performance milestone was reached. Our complete architecture is illustrated in Figure 1.

Hyperparameters such as the batch size (i.e., 512 for the first dataset and 1024 for the second dataset) and number of epochs (i.e., 282 for the first dataset and 50 for the second dataset) were set to balance computational costs and model convergence. The final settings were determined based on experiments, domain insights, and stable validation metrics.

### 4.8. Hyperparameter Optimization

Each input sample initially had a shape of (batch, 12, 1). Here, 12 represents the total time steps (n_steps × n_length, i.e., 4 × 3), and 1 indicates the single sensor dimension after preprocessing. We utilized the Keras Tuner with a random search strategy to investigate important hyperparameters within our architecture systematically. This includes parameters for the patch-extraction branch, such as ViT-like Conv1D filters, kernel size, stride, and the number of Dense units. We also examined the number of heads in the multi-head attention mechanism, the size of the LSTM layer, dropout rates, and the overall learning rate and batch size. We examined different learning rates ($1\times 10^{-4},\;5\times 10^{-4},\;\mathrm{and}\;1\times 10^{-3}$) and batch sizes (512 and 1024). This search was run for a maximum of five trials, each trained for up to 30 epochs. During this phase, we applied partial early stopping with a patience of 10 epochs and restored the best weights whenever the validation loss improved. Once the search was completed, we retrieved the top-performing hyperparameter set based on validation accuracy. The final model employed $1\times 10^{-4}$ (0.0001) in the Adam optimizer. We kept this learning rate fixed during training. We then rebuilt the final model from these best hyperparameters. We increased the maximum training epochs, applying a second early-stopping callback with patience of 20 epochs and a ModelCheckpoint callback to save only the weights, achieving the lowest validation loss. This two-step approach (i.e., initial hyperparameter tuning followed by extended training) ensured that our datasets’ final model was well optimized while minimizing the risk of overfitting. The optimal configuration identified included 256 LSTM units, a dropout rate of 0.4 for LSTM layers, a dropout rate of 0.2 for dense layers, and a learning rate of 0.001. For the ViT, we used 192 filters, a kernel size of 5, a stride of 2, 256 dense units, and four attention heads for multi-head attention. We also observed that the batch size depended on the dataset: for the larger MotionSense dataset, a batch size of 512 proved to be the most effective, while for the smaller UCI HAR dataset, we used a batch size of 1024.

Using a 1D convolution filter with 192 channels, a kernel size of 5, a stride of 2, and padding set to “same” reduced the time dimension from 12 to 6, resulting in an output shape of (batch, 6, 192). Flattening resulted in either (batch, 6 × 192) or (batch, 1152), which was then projected to (batch, 256) through a Dense layer with a dropout of 0.3, thus creating a ViT-like patch embedding. We replicated this embedding across the original 12 steps to align with the time dimension, resulting in a shape of (batch, 12, 256). Simultaneously, the same input with the shape (batch, 12, 1) was fed into the attention submodel. We utilized a multi-head attention layer with four heads and a key dimension of 64. This layer transformed the data into a shape (batch, 12, 256) through internal dense layers, incorporating a dropout of 0.1 and layer normalization to enhance stability. Combining these two streams resulted in the shape of (batch, 12, 512), effectively integrating local patch features from the ViT path with global dependencies from attention.

A bidirectional LSTM with 256 units, a dropout of 0.4, and a recurrent dropout of 0.2 processed this fused sequence, outputting a single vector of shape (batch, 512). We used a Dense layer with 256 units and a dropout rate of 0.2, followed by a Dense classification layer sized according to the number of subjects, utilizing a softmax activation function. In our experiments, we used sparse categorical cross-entropy as the loss function and the Adam optimizer for training. The learning rate determined through hyperoptimization was 0.001 for both datasets, but the batch sizes differed due to the sizes of the datasets: 512 for MotionSense and 1024 for UCI HAR. We used an 80–20 holdout scheme for each dataset and stopped training if the validation loss did not improve after 20 epochs, applying early stopping. This approach prevented overfitting and consistently selected the best model across both datasets. The optimized hyperparameters enhanced the extraction of local patches, enabled multi-head global attention, and improved LSTM-based temporal fusion, resulting in greater accuracy compared to simpler pipelines.

## 5. Evaluation and Validation

### 5.1. Metrics

As our primary goal was subject identification for continuous authentication, we focused on classification accuracy (ψ) as the principal performance metric. Formally, we calculated the accuracy by comparing each predicted label ${\hat{\lambda }}_{i}$ with its true label $\lambda_{i}$ over all *N* samples:

(12) $$\psi =\frac{1}{N}\sum_{i=1}^{N}I\left({\hat{\lambda }}_{i}=\lambda_{i}\right),$$

where I(·) is the indicator function that returns 1 if ${\hat{\lambda }}_{i}$ matches $\lambda_{i}$, and 0 otherwise. Although the accuracy suffices for a broad overview, additional metrics can yield deeper insights into class-level behaviors. In particular, confusion matrices are helpful for quantifying the severity and distribution of misclassifications across different subjects.

### 5.2. Experimental Protocol

To assess the performance of our hybrid model, we partitioned both the MotionSense and UCI HAR datasets into training and testing splits based on subject IDs. We simulated a realistic continuous authentication scenario in which previously unseen samples appeared in the test set. During model development, we employed internal validation splits on the training portion to tune hyperparameters such as the learning rate, dropout probabilities, and number of attention heads. Once these hyperparameters were optimized, we trained the model until the early stopping criteria were met, at which point we evaluated the model on the test set to measure its final generalization performance. Early stopping ensured that we did not excessively fit the irregularities of the training data, thus reducing the risk of overfitting.

While the MotionSense dataset was trained for up to 282 epochs, we limited the UCI HAR dataset to a maximum of 50 epochs. This difference arose primarily as the UCI HAR dataset consistently converged—and displayed stable validation metrics—much earlier in practice. Empirical tests showed that further training offered minimal performance gains beyond approximately 40–50 epochs and sometimes led to overfitting. Hence, 50 epochs were considered sufficient for robust subject identification on this dataset, as well as further expediting the training process without sacrificing accuracy.

### 5.3. Results

**Figure 2:**
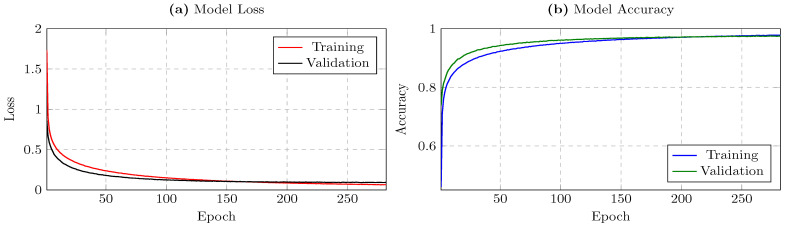
Panels (**a**,**b**) show the training vs. validation loss and accuracy, respectively, over 282 epochs for the MotionSense dataset.

We evaluated our model on the test sets for each dataset. In our experiments on the MotionSense dataset, the model ultimately achieved a validation loss of approximately 0.0942 and a validation accuracy of about 0.9751, indicating that it learned robust subject-discriminative features. The training logs (Figure 2a,b) reveal a steady increase in validation accuracy, stabilizing around 96–97% in the mid to late epochs, and a corresponding downward trend in validation loss. This behavior suggests that the model effectively captured user-specific motion signatures with minimal overfitting. The early stopping mechanism further refined this process by halting training at a near-optimal epoch, sparing the model from unnecessary additional iterations.

Observation of the training consistency indicated that the training and validation curves tracked closely after roughly 20 epochs, affirming that the learned parameters generalized well. While minor fluctuations are typical in real-world time-series classification, the final performance remains stable. Whenever the validation loss shows measurable improvement, the model checkpoint callback preserves the updated weights, ensuring that we can retrieve the best iteration across the training run. As a result, even if performance fluctuates late in training, the best checkpoint with minimal validation loss is retained for testing and deployment.

**Figure 3:**
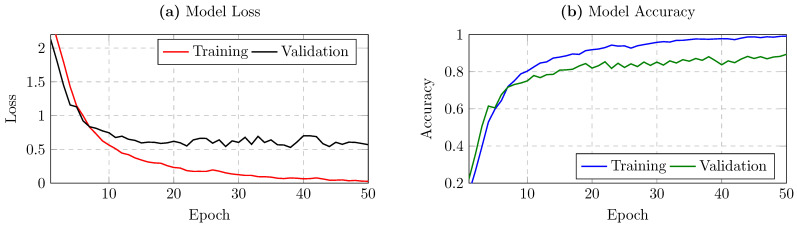
Panels (**a**,**b**) show training vs. validation loss and accuracy, respectively, over 50 epochs for the UCI HAR dataset.

For the UCI HAR dataset, we trained the model for up to 50 epochs and again applied early stopping. Figure 3a,b depict the training vs. validation loss and accuracy, respectively. Unlike the MotionSense dataset, which was run for 282 epochs, the HAR logs showed that the model typically converged within approximately 30–40 epochs, after which the validation metrics remained stable or fluctuated mildly. Consequently, extending training to 100 epochs did not yield additional improvements and sometimes risked overfitting. Thus, restricting training to 50 epochs offered a reasonable balance between computational efficiency and sufficient convergence for robust subject identification on HAR. Notably, the final test accuracy for HAR was 89.37% in our experiments, demonstrating that the model effectively generalized to new samples in this second dataset as well.

Overall, these logs and curves confirm the viability of our ViT, multi-head attention, and BiLSTM design for subject identification tasks across both MotionSense and UCI HAR. In subsequent sections, we compare the training and validation metrics in greater detail and present subject-level confusion matrices to illustrate which classes exhibit similar motion dynamics. We also highlight how each dataset’s distinct size and motion diversity influence the model’s convergence rates, thereby justifying the difference in the maximum number of training epochs used.

#### Discussion

**Figure 4:**
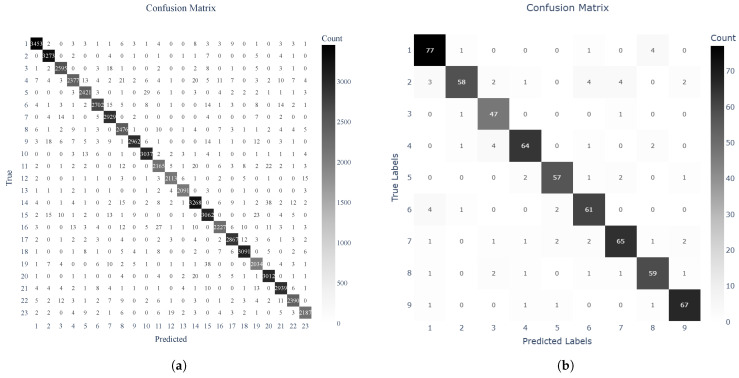
Confusion matrices for (**a**) MotionSense and (**b**) UCI HAR.

Figure 4 displays the confusion matrix for our final subject identification task, where each row corresponds to a true subject and each column corresponds to a predicted subject. Darker diagonals indicate correct classifications, while off-diagonal cells reflect misclassifications.

It can be observed that the vast majority of samples for each subject aligned with the main diagonal, confirming that the model captured individual-specific features for most participants. A few subjects appeared more frequently in off-diagonal cells, suggesting potential overlap in sensor patterns or insufficient training data for those participants. The low diffusion of misclassifications indicates that the subjects were not consistently confused. This matrix supports the quantitative results, demonstrating the model’s strong ability to differentiate unique motion signatures.

To further validate our method, we implemented a second approach from the related work—a hybrid model combining CNN and LSTM layers [15]—using the same experimental settings for 100 epochs on the MotionSense dataset. This reproduced approach ultimately attained a final loss of 0.2161 and an accuracy of 0.9295. In contrast, our proposed ViT, multi-head attention, and BiLSTM model, trained under the same conditions (100th epoch), achieved a significantly lower loss of 0.1254 and a higher accuracy of 0.9607. The reduction in loss by almost half and the accuracy improvement of over 3.12% (from 92.95% to 96.07%) demonstrate the superior efficacy of our hybrid architecture in extracting and leveraging user-specific motion signatures. In particular, the vision-transformer-like patching and multi-head attention capture local and global dependencies more effectively, while the BiLSTM component allows for robust sequence modeling. These results confirm that our solution outperformed the CNN and LSTM baseline in subject identification tasks on the MotionSense dataset, underscoring its value for continuous authentication scenarios.

Regarding computational cost, the MotionSense dataset required roughly 570 s per epoch (about nine to ten minutes) on our setup, resulting in a total training duration of over 79 h when training for the entire 282 epochs. In contrast, the UCI HAR dataset trained much faster, typically taking about one to two seconds per epoch, adding up to only a couple of minutes for 50 epochs. These time estimates can vary depending on hardware and system load. At the inference time, the model processes each sample considerably faster than during training—on the order of milliseconds per sample (the model processes each sample in approximately 2–3 milliseconds on our hardware)—making it suitable for real-time or near real-time continuous authentication scenarios. While training durations may be lengthy, the result is a robust hybrid model that accurately identifies subjects, making occasional offline training a worthwhile trade-off for long-term security.

We compared our proposed hybrid model with several baseline approaches—three traditional machine learning methods (logistic regression, KNN, and naive Bayes) and four deep learning architectures (an LSTM with 64 neurons, a CNN, a Transformer model, and an Informer model). The objective was to ensure that each comparison used the same training–testing splits. This side-by-side evaluation highlights the performance benefits of our hybrid architecture. As shown in Table 3, in the MotionSense dataset, KNN and the LSTM achieve relatively high accuracies (about 93.8% and about 93.7%, respectively), while the CNN obtains about 92.5%, the Transformer achieves 91.77%, and the results of Informer, naive Bayes, and logistic regression are comparatively lower (about 67.19%, 38.7%, and 57.4%, respectively). Our hybrid model typically outperforms these baselines, with an accuracy of 97.51%. In UCI HAR, logistic regression leads among the machine learning baselines (about 82.3%), while Informer provides strong deep learning results (about 83.7%). The CNN achieves about 80.2% accuracy, and the LSTM obtains about 76.5%. Our hybrid model again outperforms these baselines with an accuracy of 89.37%.

These findings emphasize that, although single architectures can perform reasonably well, the collaboration offered by our hybrid design can provide further gains in complex, multi-subject classification settings. In summary, the combined evidence from the training logs, performance evaluations, and confusion matrices confirm the effectiveness of the hybrid approach in meeting our continuous authentication objective. These findings open avenues for real-world applications, where rapid, accurate user identification can be maintained from sensor data collected in a variety of natural settings.

We chose a ViT-inspired approach for patch extraction because it effectively divides smartphone sensor data into small, focused time frames. This method breaks down raw time-series signals into clear and manageable segments, allowing the model to detect short-term patterns, such as micro-movements or small changes in orientation, that define user behavior. By isolating these segments explicitly, the model can ensure that critical localized patterns, often unique to specific users, are preserved and emphasized rather than weakened by longer-range aggregation typical of architectures like informer or standard time-series transformers. Models such as Informer and the time-series transformer outshine at handling extremely long time series (hundreds or thousands of timesteps) by leveraging sparse-attention mechanisms to mitigate computational complexity. By contrast, our continuous authentication setup processes relatively brief sensor windows (tens to a few hundred timesteps), so the performance benefits of specialized long-sequence optimizations are less evident. The experiments revealed that patch extraction helped to capture subtle local micro-patterns in user motions. In addition, the patch-based strategy of ViT naturally facilitates the extraction of local subsequences that are particularly relevant to smartphone sensor data. This is particularly valuable for motion-sensor inputs (e.g., accelerometer and gyroscope), where user-specific signatures manifest in transient, fine-grained fluctuations. By embedding each patch into a vector representation, the framework captures global sequence patterns (through multi-head attention) and local subsequence dynamics (through patch embeddings).

**Figure 5:**
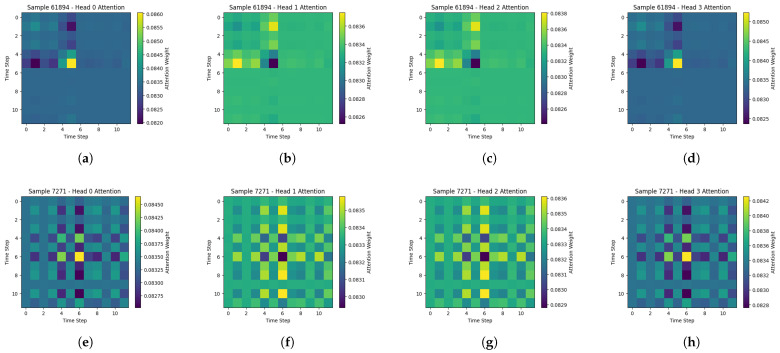
Attention heatmaps for the first sample (heads 0 (**a**), 1 (**b**), 2 (**c**), and 3 (**d**)). Warmer colors indicate higher attention weights between pairs of timesteps, suggesting that the model prioritizes short bursts of user motion (around timesteps 4–6) and attention heatmaps for the second sample (heads 0 (**e**), 1 (**f**), 2 (**g**), and 3 (**h**)). The checkerboard-like patterns indicate a more distributed allocation of attention across the window, highlighting repeated or periodic movements the model considers obvious for identifying this user.

To analyze the interpretability of our multi-head attention mechanism, we generated heatmaps illustrating the attention weight matrices for two randomly selected test samples. Each sample was fed into our model, and the resulting attention matrices (i.e., one per attention head) are represented as 12 times 12 heatmaps, as shown in Figure 5. In these diagrams, the x- and y-axes denote the 12 time steps (or patches) in our input window, while the color gradient encodes the magnitude of attention. Thus, brighter or darker cells highlight which time steps receive greater emphasis when predicting the user’s identity. For the first sample shown in Figure 5a–d, we observe that heads 0 and 3, in particular, allocate higher weights to time steps around 4 to 6, forming a visible hotspot in the upper-left region of the attention matrix. This pattern suggests that the model reacts to subtle motion signals (e.g., short bursts of acceleration) that might signify the user’s gait or micro-movements. Meanwhile, heads 1 and 2 also exhibit raised attention in that interval but distribute their focus slightly differently, implying that each head captures unique but complementary segments of the sensor data.

A contrasting picture emerges with the second sample shown in Figure 5e–h, whose attention maps display more checkerboard-like patterns. In these matrices, attention often distributes itself more evenly, with notable peaks near the midpoint (time steps 5–7). These variations could indicate periodic or repeated gestures characteristic of the user’s motion signature. As with the first sample, each attention head highlights overlapping time steps but with differing assertiveness, reinforcing that multi-head attention can separate different aspects of the time-series data. These heatmaps demonstrate that multi-head attention does not function purely as a black box. Instead, each head focuses on distinct portions of the time-series window, potentially capturing user-specific motion characteristics, such as transitions between walking and standing or small orientation shifts of the device. By combining these complementary viewpoints, the model achieves improved classification accuracy, aligning with our results that outperform simpler baselines.

While our main focus was algorithmic performance, translating these findings into real-world continuous authentication requires additional considerations. First, device resource usage (e.g., CPU, memory, and battery overhead) must be measured in an actual deployment. Second, usability and user acceptance are crucial. Continuous authentication systems should work smoothly without interrupting users’ interactions with their devices. A user study should check how intrusive these systems are, how often they wrongly lock users out, and the overall satisfaction with using smartphones. Data privacy is important because constantly monitoring motion sensors can reveal personal information. To protect information, we can use federated learning in the future or process data on devices to limit raw data transfer. We must also implement user consent options and clear data handling policies to comply with privacy laws. These actions focused on users are key to improving our system so it works well on different devices and with various user behaviors. Finally, we must combine technical testing with real-life user feedback to ensure that continuous authentication methods are effective in everyday mobile use. By combining local computation, transparent policies for data handling, and robust anonymization techniques, our approach can be integrated into real-world mobile devices.

## 6. Conclusions

This study presents a hybrid architecture that integrates ViT-inspired patch extraction, multi-head attention, and BiLSTM layers for continuous authentication using time-series data. Empirical evaluations on the MotionSense and UCI HAR datasets demonstrated a notable improvement in subject recognition accuracy, confirming the framework’s robust ability to differentiate user identities. The findings show that wearable sensor data have unique motion signatures that can be used to reliably distinguish users. Such signatures are promising for the development of real-time, unobtrusive authentication methods. Refining the patching strategy or incorporating positional embeddings from the vision transformer domain could enhance the expressiveness of features. Attention-visualization analyses confirm that our hybrid pipeline captures short-range motion features and effectively integrates them with a global context, thereby improving classification accuracy. This framework offers a basis for secure and user-specific mobile computing applications. In future work, we will test our hybrid model on real devices. We will focus on three key areas: power use, memory requirements, and inference speed. We will also conduct studies to determine how well users accept continuous sensor tracking in terms of privacy and usability. As an important next step, we will expand the sensor set, such as a magnetometer or wearable device, to identify whether richer data streams can improve classification accuracy without sacrificing resource efficiency. Finally, we aim to integrate our approach within multi-device ecosystems (e.g., smartphones and smartwatches) and implement federated or privacy-preserving techniques that minimize raw data sharing across devices. Through these tangible directions, we aim to bridge the gap between a proof-of-concept framework and a fully deployable continuous authentication system in everyday settings.

## Figures and Tables

**Table 1:** Comparison of related studies and our proposed method.

Reference	Data / Features	Classification	Method	Strengths (+) and Limitations (−)
Sagbas and Balli [14]	59 participants; 125 features (accel., gyro, typing)	Multi-class (59 distinct users)	RF, kNN, SLR + CFS (feature selection)	+ Up to 93% accuracy; very fast (0.03 ms). − Accuracy is moderate compared to modern deep learning (which can be more than 93%). Classical ML may miss subtle temporal/contextual cues.
Alzahrani et al. [15]	Accelerometer, gyroscope data (raw sensor streams)	Multi-class	CNN and BiLSTM (no patching or advanced attention)	+ Significant improvement over classical ML; integrated deep approach. − No specialized segmentation or multi-head attention; may underutilize key sensor segments if user activities vary.
Centeno et al. [20]	Public human activity dataset; Siamese embeddings	Binary (pairs of sequences)	Siamese CNN (measures similarity between motion samples)	+ Up to 97.8% accuracy; robust to frequency/length changes. − Pairwise verification less scalable for large multi-class user sets.
Abuhamad et al. [21]	Behavioral data: accel., gyro, magnetometer	Binary (one user vs. others)	Deep pipeline (implicit user patterns)	+ 98% F1-score, low rejection. − Not multi-class; only legitimate vs. others approach.
Mekruksavanich and Jitpattanakul [8]	Accel., gyro, magnet. for activity-based auth	Binary (owner vs. impostor)	Deep learning classifiers (no advanced attention)	+ Effective multi-sensor integration. − No multi-class discrimination among multiple valid users.
Cao et al. [22]	Gait data from smartphone sensors	Multi-class (user identification)	Hybrid CNN and LSTM (for gait patterns)	+ Higher performance than classical gait methods. − Focus on gait alone; may ignore other daily user activities.
Centeno et al. [23]	Smartphone sensors; autoencoder-based	Binary (anomaly detection)	Deep autoencoders (legit vs. fraudulent)	+ High accuracy, minimal overhead. − Framed as anomaly detection, not a full multi-class user ID.
Wang et al. [24]	Multimodal (touch and motion); CNN–LSTM–Attention	Multi-class	Memory-efficient CNN–LSTM–Attention (no multi-head or patch-based)	+ Robust performance combining multiple data sources. − Limited segmentation detail; lacks advanced multi-head attention for local/global dependencies.
Li et al. [25] (SNNAuth)	Accel., gyro + spiking neural networks (SNNs)	Binary (one user vs. impostor)	SNN-based energy-efficient approach	+ Low power usage, high discrimination. − Not designed for multi-user classification; focus on single user.
Shen et al. [26]	Multimodal sensor data (CNN and LSTM), final one-class classifier	One-class (legitimate vs. impostor)	CNN–LSTM and deep one-class (DeSVDD style)	+ Accurate single-user verification (multiple data sources). − Does not handle multi-subject classification; no patch-based attention.
Yantao et al. [27] (CAGANet)	CNN-extracted features and CWGAN for augmentation	One-class (OC-SVM, LOF, IF, EE)	Data augmentation (Wasserstein GAN); CNN feature extraction	+ Robustness via CWGAN; strong performance for single-user profile. − Not multi-class ID; verifies “one user vs. all” scenario only.
Muaaz et al. [28]	Accel., gyro, magnet.; 6 daily activities and 5 phone positions	Multi-class	Classical ML (time+frequency features); variable usage context	+ Covers diverse real behaviors and phone positions; multi-class. − Conventional feature engineering; only well-known classifiers, no end-to-end deep approach.
Sánchez et al. [29]	Behavioral data from multiple devices (smart office)	Multi-device (multi-class context)	Machine/deep learning with privacy focus	+ Privacy-preserving analytics across heterogeneous devices. − Less emphasis on specialized time-series extraction for single smartphone sensors.
Cariello et al. [30] (SMARTCOPE)	Accel., gyro for change-of-possession (grab, give, rest)	Event-based trigger (then re-auth)	Detect short, discrete transitions and re-auth invocation	+ Reduces false prompts, shortens attacker window by event-driven checks. − Focus on discrete transitions; no advanced segmentation/attention for continuous or subtle behaviors.
Lee and Lee [31]	Behavioral signals (accel., gyro), private dataset	Implicit continuous	Machine learning (∼98.1% accuracy)	+ High background auth accuracy; user convenience. − Smaller private dataset, simpler ML pipelines.
Our Proposed Hybrid Approach	Time-series data (advanced patch extraction, multi-head attention, BiLSTM)	Multi-class (user ID)	Patch-based segmentation and multi-head attention and BiLSTM	+ 97.51% on MotionSense; 89.37% on UCI HAR; outperforms Transformer, Informer, CNN and LSTM. − More complex architecture (i.e., higher computation).

**Table 2:** Essential information for the two datasets employed.

Dataset	Participants	Activities	Features	Sampling Rate	Notes/Window Setup
MotionSense	24 (14 M, 10 F)	6	12	50 Hz	15 trials per participant ^a^
UCI HAR	30 (ages 19–48)	6	561 ^b^	50 Hz	2.56 s windows (50% overlap)

^a^ One participant (ID 7) was excluded due to insufficient data, leaving 23 subjects. ^b^ Alternatively, raw accelerometer and gyroscope signals can be processed.

**Table 3:** Comparison of our model and baseline models on MotionSense and UCI HAR datasets.

Model	MotionSense Accuracy	UCI HAR Accuracy
Logistic regression	0.5743	0.8229
K-nearest neighbors	0.9384	0.8100
Naive Bayes	0.3866	0.3060
LSTM	0.9374	0.7649
CNN	0.9251	0.8019
Informer	0.6719	0.8374
Transformer	0.9177	0.7939
**Our Hybrid Model**	**0.9751**	**0.8937**

## Data Availability

No new data were created or analyzed in this study. Data sharing is not applicable to this article.
